# Medical Administrators: A Lesson from the Poor Law

**Published:** 1913-07-26

**Authors:** 


### PRESENT AND FUTURE OF THE POOR-LAW INFIRMARY.

### Medical Administrators: A Lesson from the Poor Law.

By H. B. M.

Specialisation is one of the features of the twentieth century. Under economic stress this division of labour is becoming finer and finer. The zenith of the specialist is especially marked in the case of the medical profession. The days of the Jonathan Hutchinson type are over (speaking of this great light purely from the point of view of his all-round excellence in the eighties). These are the days of the serologist, the protozoologist, the technician, the medical administrator.

Medical administrators have evolved as the result of the present-day system of medical treatment in large institutions, and as a natural corollary to the public awakening to the value of hygiene. Administrative specialisation can either be in the direction of public health or in the efficient management of a medical building. The medical officers of health have already proved their worth to municipalities and county authorities. The second type of medical administrator is also slowly but surely winning the confidence of a prejudiced and ignorant laity. He is fast improving his position and his status.

The position differs in London and in the provinces. In London the permanent medical superintendent is, as a rule, only to be found in Poor-Law infirmaries. There, in addition to his administrative duties, much surgical and some medical work devolves on him. He consults with his subordinate colleagues on the difficult cases.

In Scotland, however, a permanent medical superintendent is appointed as head of the large general voluntary hospital. His duties are purely administrative. The medical and surgical work falls to the lot of the residents and the specially appointed surgical and medical staff (unpaid). The salary is a fixed one, but consulting work in hospital building and management is usually allowed. He does no surgical work.

In London the present vogue is not a permanent medical superintendent but a permanent lay secretary. An annual appointment of a resident medical officer at a small salary is then made as a sort of assistant temporary medical administrator to the permanent lay administrator. As a result the institution loses the mature experience of a medically trained man with business aptitude, who has made the institution his home, and by reason of his interest and increasing knowledge can effect many economies. The London boards are evidently prepared to place greater reliance on a permanent lay secretary than on a medical superintendent. Such an appointment is usually founded on the plea of economy and also on a popular delusion that a lay secretary is a more energetic collector of subscriptions. Both of these views are erroneous.

There can be no doubt that the Scottish system is the best, indeed the ideal one. The administration of, say, such an institution as the Western Infirmary in Glasgow, under such a well-known head as Dr. D. J. Mackintosh, compares favourably with that of any of the London voluntary hospitals. Perfect administration is not necessarily economical. Its aim must be efficiency with economy. Dr. Mackintosh is now known all over the British Isles, not only as a great expert in hospital administration, but he also enjoys a large consultative practice as adviser to hospital architects. His position is a permanent one with a fixed salary, but he is permitted consultative work of this nature. After a *régime* of this character, the directors of the Western Infirmary, it may safely be anticipated, would never adopt the system now obtaining in London.

There are many suggestive indications that hospital authorities in the Metropolis will revert to the better scheme of a medically trained permanent administrative head. Only such an administrator can appreciate properly the requirements, immediate and remote, of the consulting and resident staff and the needs of the institution generally. The medical superintendents of Poor-Law infirmaries have excellent opportunities of forcing the recognition of efficient medical administrators. Reports on their institutions should be published annually. Experience of the economic management of a large Poor-Law infirmary would specially fit those who have specialised in medical administration for the position of head of a large voluntary hospital. Medical men as medical officers of health and as Poor-Law superintendents have already won their spurs and proved their worth, reliability, and business capacity in the speciality of administration, and it is time that the excellent position of governing a large voluntary hospital be within the reach of those medical men who, by their proved capacity, institutional experience, and business aptitude and training, have specially qualified themselves for such posts. Such valuable and prized positions would stimulate interest in, and be a further incentive to, this interesting branch of medicine.

